# New Perspectives on Cholesterol and Lipoprotein Metabolism

**DOI:** 10.3390/ijms241411298

**Published:** 2023-07-11

**Authors:** Bart De Geest, Mudit Mishra

**Affiliations:** Centre for Molecular and Vascular Biology, Catholic University of Leuven, 3000 Leuven, Belgium; drmuditm@gmail.com

In animals, cholesterol is an essential component of every cellular membrane and is required for cell membrane integrity. Cholesterol is insoluble in water but is solubilized by phospholipids in both membranes and plasma lipoproteins. The content of cholesterol in the cellular membranes of animals increases along the secretory pathway, being very low in the endoplasmic reticulum, higher in the Golgi apparatus, and highest in the plasma membrane [1]. Cholesterol comprises 20–40 mol% of the lipids in the plasma membranes of animals and has a major impact on the biophysical properties of lipid membranes [2]. It decreases membrane permeability to water, ions, small neutral molecules such as glucose, and gases such as oxygen by rendering these membranes more compact [2]. Cholesterol is a key player in the formation of lipid rafts, which are regions of the membrane that consist of densely-packed cholesterol, sphingomyelin, and glycosphingolipids. These liquid-ordered domains exhibit less fluidity than the surrounding plasma membrane. Lipid rafts are essential for most membrane-associated signaling cascades [3]. Mammalian cells can acquire cholesterol from de novo biosynthesis or via the uptake of exogenously derived cholesterol present in circulating low-density lipoproteins (LDL). Cholesterol is a precursor of steroid hormones. Serum cholesterol levels are tightly regulated and are physiologically present within a relatively narrow range. On the one hand, inborn errors of cholesterol synthesis cause a broad spectrum of pathological effects. On the other hand, the accumulation of cholesterol in the vascular wall in subjects with hypercholesterolemia is a cardinal feature of atherosclerosis and was demonstrated in 1910 by the German chemist and Nobel Prize winner A. Windaus in studies on human atherosclerotic lesions.

Defective cholesterol biosynthesis secondary to a decreased activity of 7-dehydrocholesterol reductase causes the Smith–Lemli–Opitz syndrome [4]. This autosomal recessive syndrome is characterized by multiple congenital malformations and behavioral and cognitive abnormalities. A deficiency of the enzyme sterol C5-desaturase, which catalyzes the second last step in cholesterol biosynthesis, namely the conversion of lathosterol to 7-dehydrocholesterol, gives rise to lathosterolosis [5]. This extremely rare syndrome is also inherited in an autosomal recessive manner and is characterized by facial dysmorphism, congenital anomalies, developmental delay, and liver disease.

As already pointed out, cholesterol plays a critical role in the assembly of membrane microdomains, whereby the activities of many signaling molecules at the plasma membrane will be modulated by the enrichment or deprivation of plasma membrane cholesterol [6]. Morinaga et al. [6]. reviewed the role of membrane cholesterol levels in the activation of the Src-family kinase Lyn following cell detachment.

Niemann–Pick intracellular cholesterol transporter 2 (NPC2) proteins are present in all vertebrates and are essential for maintaining intracellular cholesterol homeostasis. The export of LDL-derived cholesterol from late endosomes/lysosomes depends upon the cooperation of the integral membrane protein, Niemann–Pick intracellular cholesterol transporter 1 (NPC1), and the soluble protein NPC2 [7]. Hepatic stellate cells play a crucial role in hepatic fibrosis. In the presence of chronic liver damage, hepatic stellate cells transdifferentiate from a quiescent to an activated form, leading to collagen deposition in liver tissue [8].

The downregulation of NPC2 in hepatic stellate cells may play a key role in liver fibrosis and cirrhosis [9]. Wang et al. [9] demonstrated that the knockdown of NPC2 in hepatic stellate cells increases cell proliferation induced by platelet-derived growth factor BB.

Cholesterol is required for embryonic and fetal development. In the embryo or fetus, endogenous cholesterol originates from de novo synthesis and exogenous cholesterol is derived from the maternal circulation involving the uptake of maternal cholesterol-containing lipoproteins by the placenta or yolk sac, followed by processing and transport to the embryo or fetus [10]. Molecular mechanisms of this transport are only partially understood [10]. In this issue, Kallol et al. [11] present data suggesting that apolipoprotein (apo) A-I/ATP-binding-cassette-transporter-A1 (ABCA1)-dependent cholesterol transport is involved in cholesterol transport to the mother rather than in transfer to the fully developed fetus [11].

High-density lipoproteins (HDL) (density 1.063–1.21 g/mL) are circulating multimolecular platforms that exert divergent functions including reverse cholesterol transport, anti-inflammatory effects, anti-oxidative effects, immunomodulatory effects, and endothelial-function-enhancing effects [12,13]. Despite more than seven decades of research on the biochemistry, physiology, and metabolism of HDL, the exact role of HDL in atherosclerotic vascular disease still remains unclear. CSL112 is a formulation of human apo A-I in which apo A-I is complexed with phosphatidylcholine [14]. The apo A-I Event Reducing in Ischemic Syndromes II (AEGIS-II) trial investigated the impact of CSL112 (four intravenous infusions of 6 g CSL112 at one-week intervals) versus placebo (albumin solution) in 17,400 patients with acute myocardial infarction on the composite primary endpoint of cardiovascular death, non-fatal myocardial infarction, and non-fatal stroke during the high-risk 90-day period following a heart attack [15]. The estimated study completion date is November 2023. The results of this trial may clarify whether or not an HDL-targeted therapy has a clinically significant impact on atherothrombosis. In this Special Issue, Wong et al. [16] discussed pre-clinical, epidemiological, and clinical data, demonstrating the numerous roles of HDL in diabetes and in diabetes-induced complications. At present, other therapeutic areas for HDL-targeted therapies that are not directly related to atherosclerotic vascular diseases are being investigated. HDL modulate angiogenesis in a context-specific manner via distinct classical signaling pathways, resulting in an increase in hypoxia-induced angiogenesis on the one hand, and a suppression of inflammation-driven angiogenesis on the other hand [17]. In this Special Issue, two novel genes, cyclic-adenosine-monophosphate-response-element-binding protein 3 regulatory factor (CREBRF) and tripartite motif-containing protein 2 (TRIM2), that were upregulated at the mRNA level by reconstituted HDL (rHDL) were identified via the application of a microarray approach [18]. The knockdown of TRIM2 weakened endothelial cell tubulogenesis in vitro under conditions of both hypoxia and inflammation [18]. HDL-targeted therapies have also been investigated for the treatment of heart failure. Multiple independent studies have demonstrated that HDL exert direct effects on the myocardium, which are completely independent of any effects on epicardial coronary arteries [12,13]. Treatment with apo A-I nanoparticles exerts a direct positive lusitropic effect on the myocardium, suppresses the development of cardiac hypertrophy, inhibits interstitial and perivascular myocardial fibrosis, augments capillary density in the myocardium, and prevents the occurrence of heart failure or reverses established heart failure [12]. In this Special Issue, Mishra et al. [19] described a new murine model of heart failure with preserved ejection fraction (HFpEF) induced by feeding female C57BL/6N mice coconut oil for 26 weeks. An intervention with apo A-I_Milano_ nanoparticles (MDCO-216) resulted in a normalization of cardiac function and a significant amelioration of exercise capacity. These positive results have subsequently been confirmed in a murine model of hypertension-associated HFpEF induced by a subcutaneous infusion of angiotensin II in combination with 1% NaCl in drinking water [20].

Cholesterol and phospholipids are the two major lipids within the cell membrane. Arachidonic acid is a polyunsaturated fatty acid (C20:4 (ω-6)) present in phospholipids. An integrated perspective on the key role of this ω-6 fatty acid and its metabolites in the initiation and progression of obesity, diabetes, non-alcoholic fatty liver disease, and cardiovascular diseases was provided by Sonnweber et al. [21]. Lipid and immune pathways are crucial in the pathophysiology of these diseases. In this respect, arachidonic acid and its derivatives connect nutrient metabolism to immunity and inflammation [21]. The importance of nutrient metabolism is also illustrated by the beneficial effects of icosapent ethyl, i.e., ethyl eicosapentaenoic acid, in the REDUCE-IT trial [22]. The administration of this ω-3 fatty acid had a very marked impact on the occurrence of major adverse cardiovascular events [22].

There are seven members of the LDL receptor gene family and three of these members have a major role in lipoprotein metabolism: the LDL receptor, the very low-density-lipoprotein receptor (VLDL receptor), and the LDL receptor-related protein 1 (LRP1). LRP1 was originally identified as an endocytic receptor for α_2_-macroglobulin–proteinase complexes and apo E but has subsequently been shown to be a multifunctional transmembrane receptor that can bind a plenitude of unrelated ligands with high affinity [23]. Insulin stimulates LRP1 trafficking to the plasma membrane, which results in increased LRP1 cell surface levels and functional activity [23]. This leads to an increased postprandial clearance of chylomicron remnants [24]. The interaction between LRP1 and insulin is bidirectional since LRP1 directly regulates the insulin signaling pathway by regulating subcellular receptor tyrosine kinase trafficking [25]. In this Special Issue, Actis Dato and Chiabrando [26] discuss the role of LRP1 in insulin receptor trafficking and intracellular signaling activity, and the impact of LRP1 on the regulation of glucose homeostasis in adipocytes, muscle cells, and in the brain. They also discuss the modulation of inflammation by LRP1. Familial hypercholesterolemia is an autosomal codominant disorder with a prevalence of 1:250 and is characterized by increased serum levels of pro-atherogenic lipoproteins. This disorder is most often caused by mutations in the LDL receptor, and more than 3000 variants have already been reported. The genetics, diagnosis, and treatment of familial hypercholesterolemia were discussed in a comprehensive review by Benito-Vicente et al. [27] in this Special Issue. The discrimination of pathogenic and non-pathogenic mutations of the LDL receptor requires methodologies that allow for the quantification of LDL receptor activity in vitro. This specific topic is discussed in a very systematic way in this Special Issue by Benito-Vicente et al. [28]. Most subjects with hypercholesterolemia suffer from polygenic or multifactorial hypercholesterolemia. Genetic traits that may contribute to the susceptibility of developing hypercholesterolemia and also to the interindividual differences in statin response are investigated in the study of Angelini et al. [29].

Proprotein convertase subtilisin/kexin type 9 (PCSK9) promotes LDL receptor degradation, leading to increased concentrations of pro-atherogenic lipoproteins. Fully human monoclonal antibodies directed against PCSK9, namely alirocumab and evolocumab, have been used in the treatment of selected patients with hypercholesterolemia for several years [30,31]. Jiang et al. [32] applied the scrambled disulfide bond technique to generate conformationally-altered isomers of the catalytic domain of mouse PCSK9 and selected four immunogens. Their immunotherapy strategy produced a marked immune response against native murine PCSK9 in C57BL/6J and apo E-deficient mice. This resulted in a significant decrease in plasma cholesterol levels in C57BL/6J mice and to a lesser extent in apo E-deficient mice.

In summary, this Special Issue covers a broad range of topics with a high degree of biochemical, physiological, and clinical relevance in the field of cholesterol and lipoprotein metabolism.
